# A Novel *Bmal1* Mutant Mouse Reveals Essential Roles of the C-Terminal Domain on Circadian Rhythms

**DOI:** 10.1371/journal.pone.0138661

**Published:** 2015-09-22

**Authors:** Noheon Park, Hee-Dae Kim, Solmi Cheon, Hansang Row, Jiyeon Lee, Dong-Hee Han, Sehyung Cho, Kyungjin Kim

**Affiliations:** 1 School of Biological Sciences, Seoul National University, Seoul, Korea; 2 Department of Brain & Cognitive Sciences, Seoul National University, Seoul, Korea; 3 Department of Neuroscience & Neurodegeneration Control Research Center, Kyung Hee University, Seoul, Korea; 4 Department of Physiology, Kyung Hee University School of Medicine, Seoul, Korea; 5 Department of Brain Science, Daegu Gyeongbuk Institute of Science & Technology (DGIST), Daegu, Korea; University of Lübeck, GERMANY

## Abstract

The mammalian circadian clock is an endogenous biological timer comprised of transcriptional/translational feedback loops of clock genes. *Bmal1* encodes an indispensable transcription factor for the generation of circadian rhythms. Here, we report a new circadian mutant mouse from gene-trapped embryonic stem cells harboring a C-terminus truncated *Bmal1* (*Bmal1* ^*GTΔC*^) allele. The homozygous mutant (*Bmal1* ^*GTΔC/GTΔC*^) mice immediately lost circadian behavioral rhythms under constant darkness. The heterozygous (*Bmal1* ^*+/GTΔC*^) mice displayed a gradual loss of rhythms, in contrast to *Bmal1* ^*+/-*^ mice where rhythms were sustained. *Bmal1* ^*GTΔC/GTΔC*^ mice also showed arrhythmic mRNA and protein expression in the SCN and liver. Lack of circadian reporter oscillation was also observed in cultured fibroblast cells, indicating that the arrhythmicity of *Bmal1* ^*GTΔC/GTΔC*^ mice resulted from impaired molecular clock machinery. Expression of clock genes exhibited distinct responses to the mutant allele in *Bmal1* ^*+/GTΔC*^ and *Bmal1* ^*GTΔC/GTΔC*^ mice. Despite normal cellular localization and heterodimerization with CLOCK, overexpressed BMAL1^GTΔC^ was unable to activate transcription of *Per1* promoter and BMAL1-dependent CLOCK degradation. These results indicate that the C-terminal region of *Bmal1* has pivotal roles in the regulation of circadian rhythms and the *Bmal1* ^*GTΔC*^ mice constitute a novel model system to evaluate circadian functional mechanism of BMAL1.

## Introduction

Most living organisms harbor biological timers called circadian clocks to drive daily physiological and behavioral rhythms. In mammals, the molecular circadian clock is composed of interlocked feedback loops. The CLOCK:BMAL1 heterodimer activates the transcription of clock genes such as *Periods* (*Per1/2*), *Cryptochromes* (*Cry1/2*), *Rors* and *Rev-erbs*. The translated PER and CRY proteins subsequently repress the activity of CLOCK:BMAL1. RORs and REV-ERB proteins contribute to rhythmicity *via* the competitive regulation of *Bmal1* promoter activity [1, 2].

The clock is endowed with the intrinsic redundancy due to homologous genes encoding functionally overlapping components. For example, while *Per1* and *Per2* double knock-out mice exhibited an immediate loss of circadian rhythm in constant darkness, single gene knock-out mice retained largely normal rhythms. Interestingly, *Bmal1* is unique among the core clock genes in that its disruption alone leads to arrhythmicity [3]. *Bmal1* ^*-/-*^ mice have also been shown to display various physiological deficits including defective glucose/lipid metabolism, progressive arthropathy, early aging and decreased longevity [4–6]. Therefore, *Bmal1* ^*-/-*^ mice have been a valuable animal model for evaluating the impact of circadian rhythms on physiology and behavior. However, several lines of evidence from tissue-specific rescue experiments indicated that certain phenotypes may result from the consequences of tissue-specific, as opposed to core clock, functions of *Bmal1* and expression of its paralogous gene *Bmal2* can rescue several phenotypes of *Bmal1* ^*-/-*^ mice including circadian rhythmicity [7–9]. Taken together, various phenotypes of *Bmal1* ^*-/-*^ mice, which are not related to the rhythm itself have limited further analysis of its circadian and physiological roles.

The C-terminal region of BMAL1 plays an important regulatory function for periodic oscillation. The deletion or site-directed mutagenesis in this region leads to loss of circadian rhythms [10, 11]. Several studies demonstrated that the binding sites for transactivation factors or CRYs reside in the C-terminal region [12–14]. In particular, the G and H domains of BMAL1 have been reported as important regions for generating circadian rhythms [11]. These results raise the possibility that BMAL1 C-terminal truncation or specific mutation is sufficient to abrogate rhythms while leaving other domains intact *in vivo*.

Here, we report a new animal model for evaluating the roles of *Bmal1* in circadian rhythm and physiology. The mice carrying a C-terminus truncated *Bmal1* allele (*Bmal1* ^*GTΔC*^) were generated from gene-trapped ES cells. Although the heterozygous (*Bmal1* ^*+/GTΔC*^) and homozygous (*Bmal1* ^*GTΔC/GTΔC*^) mutant mice also suffer loss of circadian rhythms, these mice displayed significantly distinct molecular and physiological phenotypes compared with the severely compromised *Bmal1* ^*-/-*^ mice, providing a novel animal model for understanding the roles of *Bmal1* in mammalian clock functions.

## Materials and Methods

### Generation of *Bmal1* ^*GTΔC*^ mice

To generate *Bmal1* ^*GTΔC*^ mice, ES cells harboring C-terminus truncated *Bmal1* gene were obtained from Sanger Institute Gene Trap Resource (SIGTR, Cambridge, UK) [15]. The ES cells were injected into blastocystes and chimeric mice were generated as described previously [16]. The genotypes were determined by using three primer polymerase chain reaction (PCR) method (WT-F, 5’-CCTCTCCAAGGCTGTTTCTG-3’, WT-R, 5’-TGAGCCTGCCCTGGTAATAG-3’ and *Bmal1* ^*GTΔC*^-R, 5’-GGCCAAGTTTGTTTCCTTGA-3’). The mice were backcrossed for six generations on C57BL/6 mice purchased from the Orient Bio (Orient Bio Inc., Seongnam, Korea). For generating littermates, ten weeks-old male mice were paired at a ratio of 1:2 with female mice.

### Housing conditions and monitoring of wheel running activity

After genotype selections, male mice were transferred to a conventional animal facility and housed individually in Nalgene cages (Nalge Nunc, Rochester, NY). All mice were maintained in a specific pathogen free (SPF) animal facility and a sound-proof isolated room with a constant temperature (22–23°C) and humidity (50 ± 10%). Lights were on at 08:00 AM and off at 20:00 PM (L:D = 12h:12h). Illumination was provided by 32 W cool white fluorescence bulbs adjusted to 350 lux at the bottom of cage and completely blocked during the scotophase (< 0.5 lux). Mice were housed with a wood-chip bedding [17] and fed *ad libitum* with NIH-31 rodent chow (Zeigler Brothers, Gardners, PA) and tap water. All animal experiments were made to minimize suffering and approved by Seoul National University Institutional Animal Care and Use Committee. For wheel running activity experiments, the mice were transferred to a cage supplemented with a wheel running assembly at 8 weeks of age (Mini-Mitter, Bend, OR). After 1 week of adaptation, the wheel running activity was recorded at 6 min intervals and the raw data files were analyzed as described previously [18]. The fast Fourier transform (FFT) and free running period (FRP) were analyzed by ClockLab data analysis program (Actimetrics, Wilmette. IL). For the circadian tissue sampling, mice were sacrificed every 6hr from 30hr to 48hr in a constant darkness condition.

### Cell culture, transfection, and luciferase assay

NIH-3T3 and mouse embryonic fibroblasts (MEFs) were maintained in DMEM containing 10% FBS, 100 units penicillin and 100 g/ml streptomycin. For MEF preparations, 13.5 day old mouse embryos were collected and dissociated by mincing and Trypsin-EDTA treatment [19]. Transfections were performed by using Lipofectamine and Plus reagent according to the manufacturer’s instructions (Invitrogen, Carlsbad, CA). For luciferase assays, 10 ng of pmPer1 6.8Kbp-luciferase construct was co-transfected with other effectors and total quantity of DNA was adjusted by pcDNA 3.0. Cells were harvested and luciferase activities were measured by dual luciferase assay system. The transfection efficiency was adjusted by renilla luciferase activity of pRL-TK (Promega, Madison, WI). All assays were performed in triplicate and repeated three times independently.

### *In situ* hybridization

Mouse brains were immediately frozen in liquid nitrogen-chilled isopentane. Frozen sections (12mM) were cut coronally and fixed with 4% paraformaldehyde and dehydrated. Riboprobes were produced from the plasmids containing *Per2* (GenBank accession number: AF036893, n.t. 186–555) and *Bmal1* (GenBank accession number: AF022992, n.t. 1261–1614) cDNA. The probes for *Bmal1* mRNA were designed to detect both *Bmal1* ^*wt*^ and *Bmal1* ^*GTΔC*^. Antisense riboporbes were prepared by in vitro transcription using α-[^35^S] UTP as manufacturer's instruction (Promega, Madison, WI). Sections were hybridized with the riboprobes overnight at 52°C and washed with sodium-saline citrate. Then, the sections were treated with RNase A (20 mg/ml) for 30 min at 37°C, and rinsed. Radioactive signals were visualized by exposing the sections to β-max film for 7 days (Kodak, Rochester, NY).

### mRNA analysis

Mouse liver samples were immediately frozen by liquid nitrogen at the indicated times and stored at -80°C until the analysis. For mRNA analysis, total RNAs were extracted from the liver by the single step acid guanidinium thiocyanate-phenol-chloroform method. The purified RNA was reverse-transcribed according to the manufacturer’s instructions (Promega). The mRNA levels were quantified by the real-time PCR using following primer sets and normalized by GAPDH levels. Per1-F, 5′-GTGTCGTGATTAAATTAGTCAG-3′, Per1-R, 5′-ACCACTCATGTCTGGGCC-3′; Per2-F, 5′-GCGGATGCTCGTGGAATCTT-3′, Per2-R, 5′-GCTCCTTCAGGGTCCTTATC-3′; Bmal1-F, 5’-CCTAATTCTCAGGGCAGCAGAT-3’, Bmal1-R, 5’-TCCAGTCTTGGCATCAATGAGT-3’, Clock-F, 5’-TTGCTCCACGGGAATCCTT-3’, Clock-R, 5’-GGAGGGAAAGTGCTCTGTTGTAG-3’, Rev-erbα-F, 5’-AAGACATGACGACCCTGGAC-3’, Rev-erbα-R, 5’-GAGTCAGGGACTGGAAGCTG-3’, Cry1-F, 5’-CGAATGAATGCAAACTCCCT-3’, Cry1-R, 5’-AAAAATTCACGCCACAGGAG-3’, Dbp-F, 5’-CAAGAACAATGAAGCAGCCAAGAG-3’, Dbp-R, 5’-AGGGCACAAGCAACATTACC-3’, GAPDH-F, 5′-CATGGCCTTCCGTGTTCCTA-3′, GAPDH-R, 5′-CCTGCTTCACCACCTTCTTGA-3′. The probes for *Bmal1* mRNA were designed to detect both *Bmal1* ^*wt*^ and *Bmal1* ^*GTΔC*^.

### Immunoprecipitation (IP) and immunoblotting (IB)

For IB experiments, mouse liver tissues and cells were lysed with radioimmunoprecipitation assay (RIPA) buffer (50 mM Tris [pH 8.0], 1% Triton X-100, 150 mM NaCl, 1 mM EDTA, 1 mM EGTA, 1 mM phenylmethylsulfonyl fluoride, 1 mM NaF, 1 mM Na_3_VO_4_, and 1X protease inhibitor cocktail (Sigma, St. Louis, MO). Proteins were resolved on 6% or 8% SDS-polyacrylamide gels and transferred onto polyvinylidene difluoride membranes (Millipore, Billerica, MA). The membrane was blocked for 1 h at room temperature in 10% skim milk solution and incubated with several primary antibodies such as anti-BMAL1 [20], CLOCK (SantaCruz, Dallas, TX), β-GAL (Promega, Madison, WI), MYC (SantaCruz, Dallas, TX), ACTIN (SantaCruz, Dallas, TX). Immunoreactive bands were visualized with ECL reagents (Thermo Scientific, Waltham, MA) according to manufacturer's instructions. For IP experiments, NIH3T3 cells were transfected with the indicated plasmids. At 36 hours posttransfection, the cells were lysed with RIPA buffer and centrifuged at maximum speed for 20 min at 4°C. Equal amounts of total protein were incubated with 2μg of anti-Flag M2 (Sigma, St. Louis, MO) for 1.5 h at 4°C and then added protein A/G-Sepharose bead slurry. The final immune complexes were analyzed by immunoblotting with indicated antibodies [21].

### Real-time monitoring of bioluminescence

To observe endogenous circadian rhythms of *Bmal1* ^*+/GTΔC*^ and *Bmal1* ^*GTΔC/GTΔC*^, MEFs were generated from embryos of mutant mice that were bred with PER2::LUC knock-in mice [22]. At 6^th^ passage, those MEFs were incubated with 1μM dexamethasone (DEX) for 2hr, then, media were changed with a recording media containing 100μM D-luciferin (Promega). To analyze the effect of *Bmal1* ^*GTΔC*^ on the circadian rhythm by the transient transfection, immortalized wild type (WT) MEFs were transfected with mutant clones and dual-color luciferases (Per2-SLR2 and Bmal1-Eluc). After 36hr of transfection, cells were synchronized by 1μM DEX treatment and bioluminescence was measured for 1min at 10min interval using KronosDio (ATTO Corporation, Tokyo, Japan) [21]. Each luciferase activity was calculated as previously reported [23]. The period length and amplitude were calculated as previous described [21].

### Fluorescence and bimolecular fluorescence complementation (BiFC) assays

To analyze the effect of *Bmal1* C-terminal region on the localization of BMAL1 and CLOCK simultaneously, VENUS protein was fused to BMAL1^wt^ and BMAL1^GTΔC^, and CERULEAN (CERUL) to CLOCK as indicated [24]. For BiFC assay, amino acid residues 1 to 172 (VN) and 173 to 238 (VC) of VENUS protein was fused to BMAL1 and CLOCK, respectively. NIH-3T3 cells were transfected with the indicated constructs, incubated at 37°C for 36hr and visualized by using Delta-Vision fluorescence microscope (Applied Precision, Isaquah, WA).

### Statistical analysis

Data were analyzed by one-way or two-way analysis of variance (ANOVA) with Tukey post-tests using GraphPad PRISM (GraphPad Prism Software, La Jolla, CA). A *p* value less than 0.05 was considered as a significant difference.

## Results

### Generation of the *Bmal1* ^*GTΔC*^ mice

**Fig 1:**
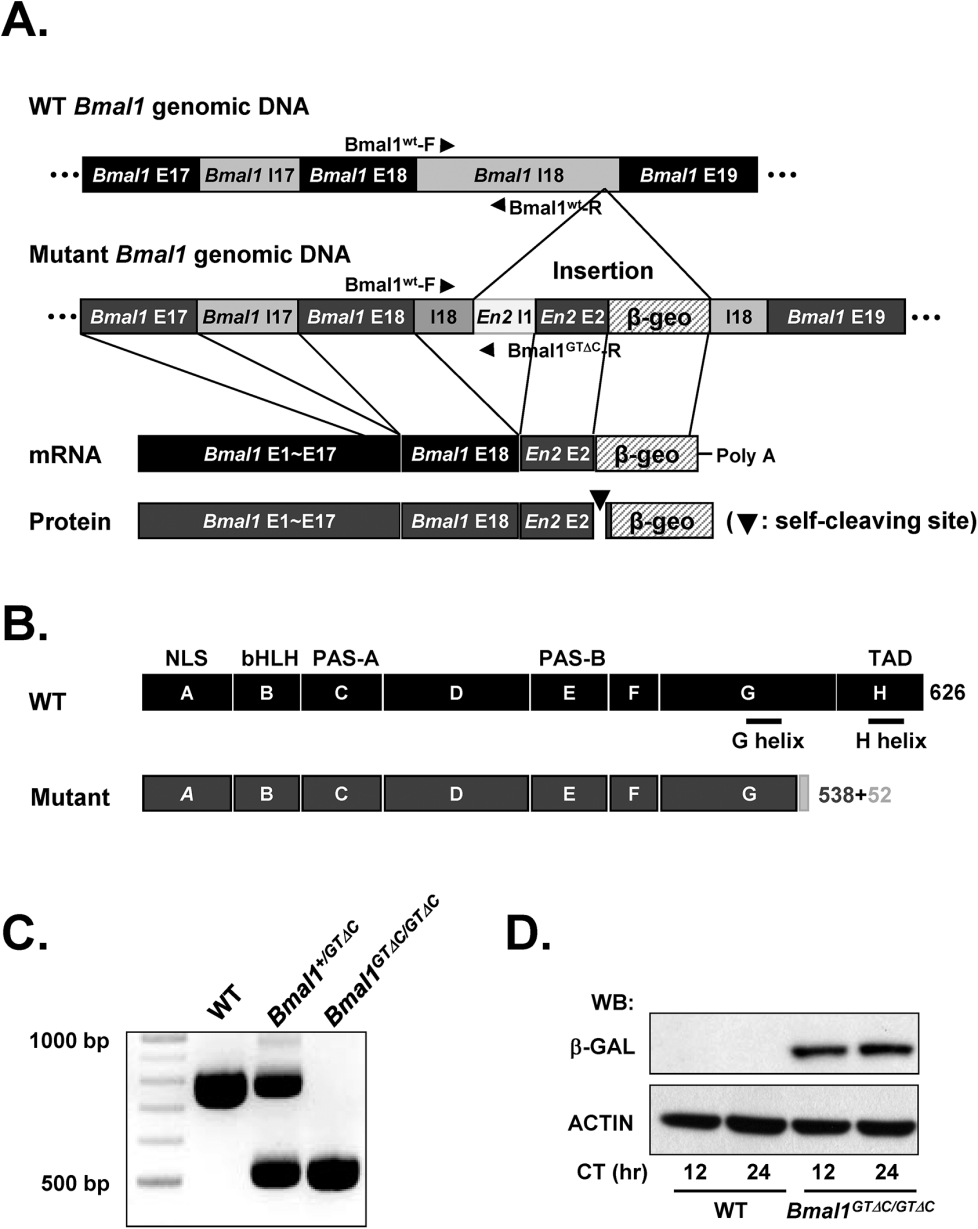
Structures and genotyping of *Bmal1* ^*GTΔC*^ mice. (A) The genomic structure of ES cells harboring *Bmal1* ^*GTΔC*^ allele. Black and grey colors indicate the exon (E) and intron (I) region, respectively. Above panel represents intact organization of Bmal1 DNA and below panel show the insertion site of the gene-trap vector. Black triangle indicates the location of the primer sequence. (B) Domains of WT and BMAL1^GTΔC^ proteins. (C) The genotypes were determined by using three primers PCR method (Bmal1^wt^-F, Bmal1^WT^-R and Bmal1^GTΔC^-R). (D) The genotypes were also determined by western blotting of β-GAL.

We obtained ES cells from SIGTR where the gene trap vector was inserted in the *Bmal1* gene locus. To identify the precise insertion site, we sequenced the 18^th^ intronic DNA region of *Bmal1* based on SIGTR sequence tag information (#CE0167, *Arntl* ^*Gt(CE0167)Wtsi*^) [15]. The gene trap vector containing *Engrailed 2* (*En2*), self-cleaving sequence, *β-Geo* (*β-Galactosidase* + *neomycin*) and poly(A) signal was found to be inserted at 3704 bp away from the 18^th^ exon (Fig 1A and S1A Fig). cDNA sequencing revealed that the 18^th^ intron of *Bmal1* ^*GTΔC*^ was successfully spliced out and the 17^th^ exon was connected to the En2 exon, self-cleaving sequence 2A and *β-Geo* to generate a functional mRNA (Fig 1A and S1A Fig). Domain structures of WT and mutant BMAL1 proteins are illustrated (Fig 1B and S1B Fig). Although the hypothetical size of the fusion protein is approximately 180 kDa, β-GALACTOSIDASE (β-GAL) antibody detected 110 kDa proteins, indicating a functional self-cleaving 2A peptide sequence (Fig 1D). The genotypes were further determined by three primers PCR and immunoblotting (Fig 1C and 1D). Collectively, these results indicated that the gene trap vector was inserted at the 18^th^ intronic region of *Bmal1* generating C-terminus truncated BMAL1 (residues 1–538) plus 52 amino acids from EN2 and the self-cleaving sequence. Using these heterozygous male and female mutant mice, we generated WT, heterozygous and homozygous mice for the experiments. The genotypes of littermates exhibited a 1:2:1 ratio, indicating no overt effect of *Bmal1* ^*GTΔC*^ allele on embryonic development (Table 1).

**Table 1:** The progeny genotypes of *Bmal1* mutant and null mice.

*Bmal1* ^*+/GTΔC*^ X *Bmal1* ^*+/GTΔC*^			*Bmal1* ^*+/-*^ X *Bmal1* ^*+/-*^		
+/+	+/*GTΔC*	*GTΔC/GTΔC*	+/+	+/-	-/-
21	60	28	27	59	30

### Altered circadian rhythms in *Bmal1* ^*GTΔC*^ mice

**Fig 2:**
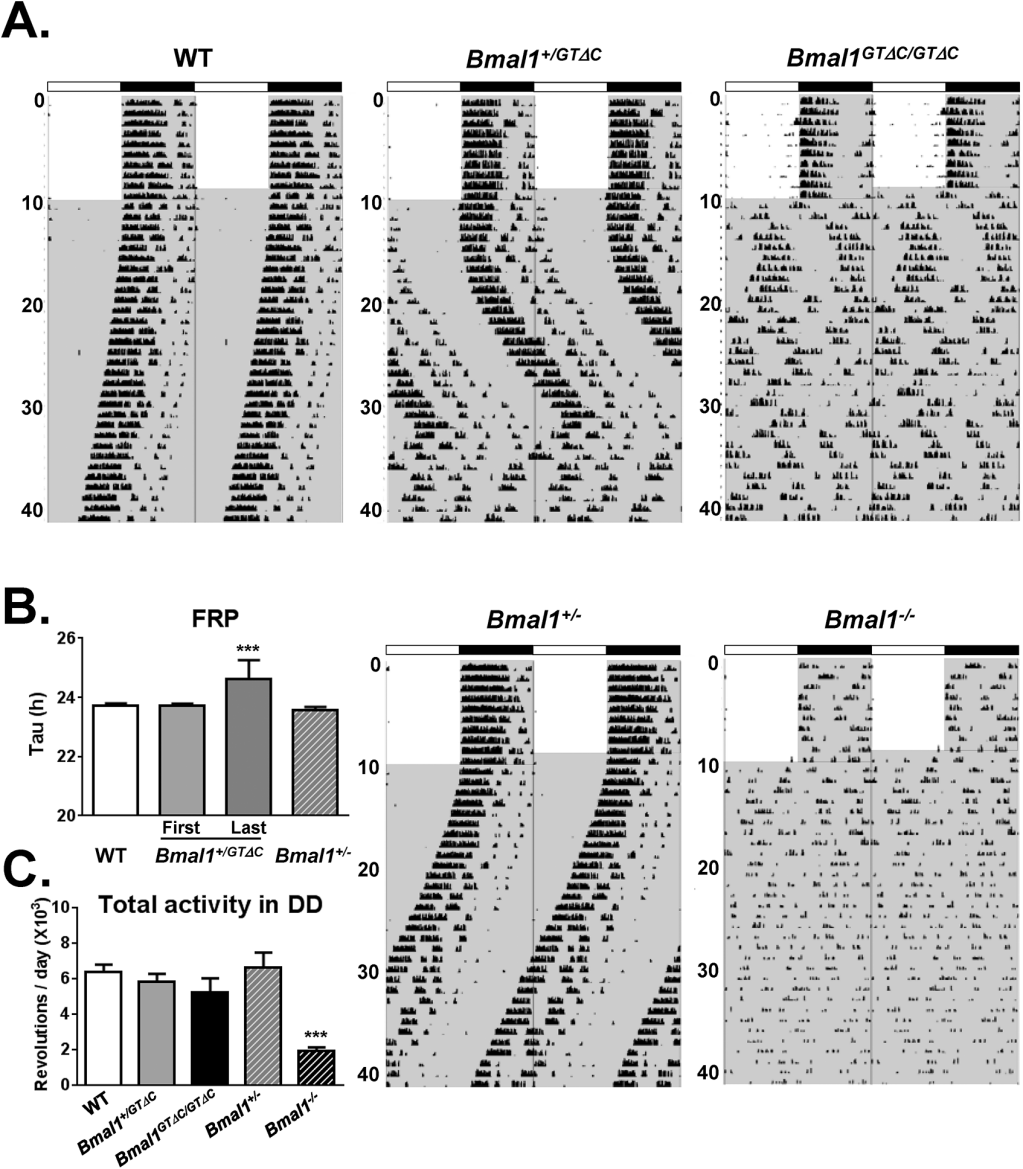
Representative double-plot actograms and locomotor activities of WT, *Bmal1* ^*+/GTΔC*^, *Bmal1* ^*GTΔC/GTΔC*^, *Bmal1* ^*+/-*^ and *Bmal1* ^*-/-*^mice. (A) The representative 40-day wheel-running actograms under 10 days of L:D = 12 h:12 h and 40 days under DD are shown. The light and dark phases are illustrated by bright fields and gray shadows within the figure, respectively. (B) FRPs of each genotype. The FRPs of WT and *Bmal1* ^*+/-*^ mice were calculated from the data of initial 10 days in DD. Those of *Bmal1* ^*+/GTΔC*^ mice were divided as two different periods. “First” indicates the initial 5 days when the mice were addressed in DD and “Last” indicates the interval of 5 days before losing their rhythms. (C) Effects of genotypes on total activity of wheel running activity. The wheel running data were collected and analyzed. One-way ANOVA along with Tukey’s post-tests was conducted for statistical evaluation. Asterisks indicate significant differences (***p<0.001) compared with WT mice. Data are represented as the mean ± S.E.M. (n = 4~8 per group).

We next examined the circadian behavior of *Bmal1* ^*GTΔC*^ mice by monitoring wheel-running activities. To directly compare with *Bmal1* null mutants, we also examined the behavior of *Bmal1* ^*+/-*^ and *Bmal1* ^*-/-*^ mice. *Bmal1* ^*+/GTΔC*^ mice showed unstable circadian periodicity for ~20 days under the constant darkness (DD) condition. Compared with WT and *Bmal1* ^*+/-*^ mice (Fig 2A), 90% (10/12) of the *Bmal1* ^*+/GTΔC*^ mice showed arrhythmic behaviors after ~20 days in DD. The remaining mice also lost their rhythms after showing extremely long FRP (S2A Fig). Initially, *Bmal1* ^*+/GTΔC*^ mice did not have significantly altered FRP during first 5 days in DD. However, their FRPs were lengthened continuously followed by rhythm loss. The FRP in the last 5 days before losing the rhythms was significantly increased (Fig 2B). However, the amplitude of *Bmal1* ^*+/GTΔC*^ mice in the last 5 days was not significantly changed (S2C Fig). These unusual circadian behaviors of *Bmal1* ^*+/GTΔC*^ mice were not found in WT and *Bmal1* ^*+/-*^ mice (Fig 2A). As expected, *Bmal1* ^*GTΔC/GTΔC*^ immediately became arrhythmic under DD. Furthermore, *Bmal1* ^*-/GTΔC*^ mice also lost behavioral rhythms (S2B Fig). Interestingly, the total locomotor activities of *Bmal1* ^*GTΔC/GTΔC*^ mice were comparable to those of WT mice, whereas *Bmal1* ^*-/-*^ mice manifested significantly decreased activities regardless of LD cycles as reported previously (Fig 1C and S2D Fig) [9]. These results strongly suggested that the truncation of BMAL1 C-terminal region is sufficient to disrupt circadian rhythm, which may involve a different mechanism compared to the null mutant mice.

### Altered mRNA and protein expression of clock genes in *Bmal1* ^*GTΔC*^ mice

**Fig 3:**
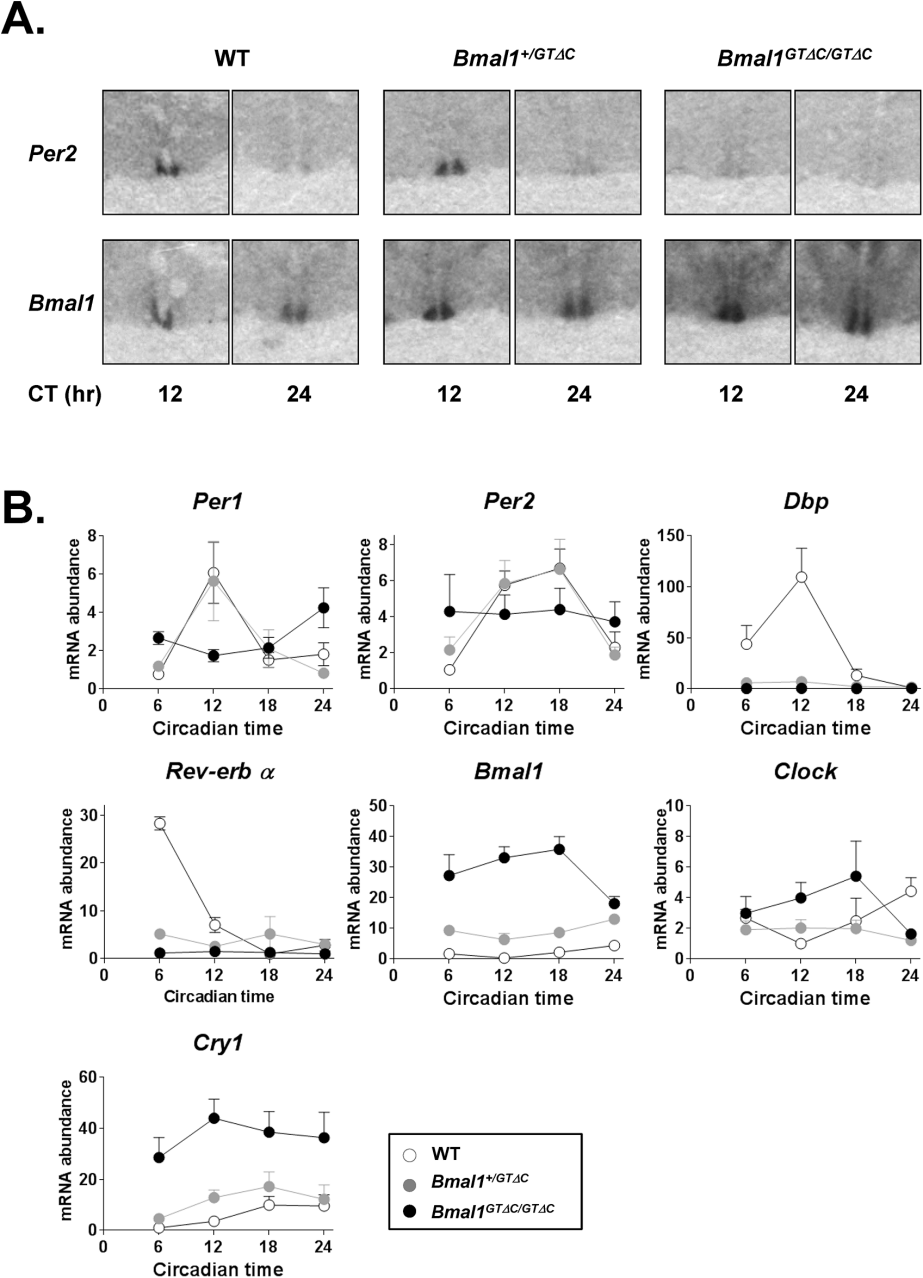
Altered circadian gene expression of WT, *Bmal1* ^*+/GTΔC*^ and *Bmal1* ^*GTΔC/GTΔC*^ mice. (A) *In situ* hybridization results of *Per2* and *Bmal1* mRNA expression in the SCN of WT, *Bmal1* ^*+/GTΔC*^ and *Bmal1* ^*GTΔC/GTΔC*^ mice. (B) The mRNA expression in the liver. Data are represented as the mean ± S.E.M. (n = 3~6 per group).

To understand the arrhythmic circadian behavior of *Bmal1* ^*GTΔC*^ mice, we examined the mRNA and protein expression profiles of clock genes. As shown in Fig 3A, *Per2* mRNA expression of WT mice showed peak expression at CT12 in the SCN, while reaching nadir expression at CT24. These patterns were also observed in *Bmal1* ^*+/GTΔC*^ mice. However, *Per2* mRNA expression was arrhythmic in *Bmal1* ^*GTΔC/GTΔC*^ mice and remained at low levels. Interestingly, although *Bmal1* mRNA expression was rhythmic in both WT and *Bmal1* ^*+/GTΔC*^ mice, greater levels were observed in *Bmal1* ^*+/GTΔC*^ mice. The increase in mRNA levels was more pronounced in *Bmal1* ^*GTΔC/GTΔC*^ mice that showed arrhythmic expression.

To further substantiate and quantify these patterns, we examined the mRNA expression profiles of several clock genes in the liver. The oscillation of *Per1* and *Per2* mRNA was abrogated in *Bmal1* ^*GTΔC/GTΔC*^ mice, but there was no difference in their rhythmic expression between WT and *Bmal1* ^*+/GTΔC*^ mice (Fig 3B). However, those of *Rev-erbα* and *Dbp* mRNA were significantly decreased in both heterozygous and homozygous mutant mice. Interestingly, the levels of *Bmal1* and *Cry1* mRNAs were increased in both *Bmal1* ^*+/GTΔC*^ and *Bmal1* ^*GTΔC/GTΔC*^ mice, whereas *Clock* mRNA expression showed no difference (two-way ANOVA with Tuckey’s post-tests, **p<0.01). Furthermore, these observed increases were coupled with decreased *Rev-erbα* mRNA expression. In light of the reduced clock gene mRNA levels in *Bmal1* ^-/-^ mice [3], these results implied that the rhythm disruption in *Bmal1* ^*GTΔC*^ mice entails a different mechanism compared with the null mutants.

**Fig 4:**
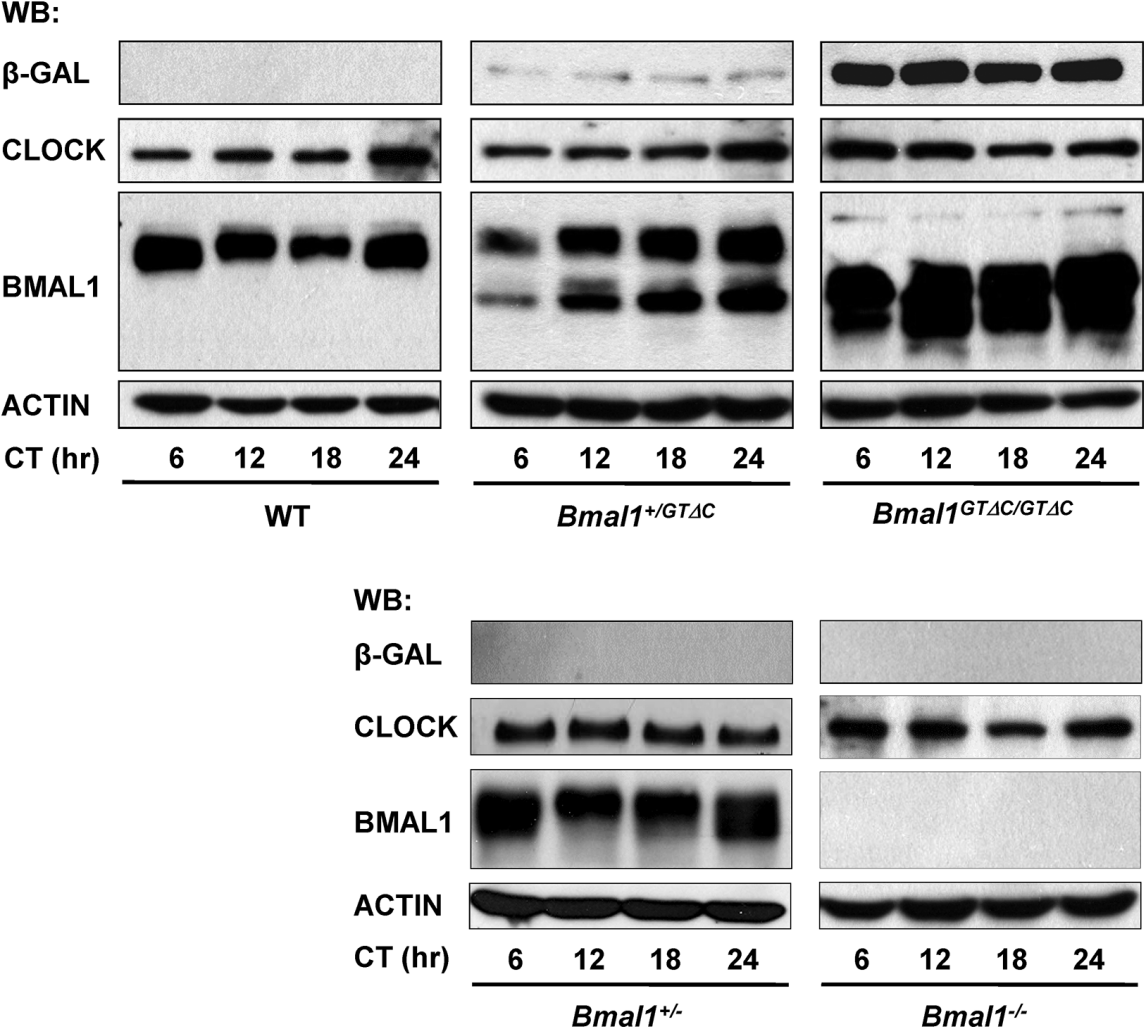
The representative protein expression profiles of the liver. Mouse liver tissues were harvested at the indicated circadian times and analyzed by western blotting. The circadian expression profiles of clock proteins in WT, *Bmal1* ^*+/GTΔC*^, *Bmal1* ^*GTΔC/GTΔC*^, *Bmal1* ^*+/—*^and *Bmal1* ^*-/-*^ mice (n = 3).

In addition to mRNA profiles, we also examined levels of clock proteins in the liver. As expected, β-GEO proteins were only found in *Bmal1* ^*+/GTΔC*^ and *Bmal1* ^*GTΔC/GTΔC*^ mice, and the size of BMAL1^GTΔC^ proteins was slightly reduced compared with BMAL1^wt^. Despite robust circadian behavior during the first several days in DD, *Bmal1* ^*+/GTΔC*^ mice did not exhibit robust oscillations of BMAL1^wt^ and BMAL1^GTΔC^ proteins compared with WT and *Bmal1* ^*+/-*^ mice. In accord with behavioral and mRNA data, there was no circadian oscillation of BMAL1^GTΔC^ proteins in *Bmal1* ^*GTΔC/GTΔC*^ mice, and no BMAL1 protein was detected in *Bmal1* ^*-/-*^ mice. In contrast to the increased *Bmal1* mRNA levels of the liver of *Bmal1* ^*+/GTΔC*^ and *Bmal1* ^*GTΔC/GTΔC*^ mice, there were only subtle changes of BMAL1^wt^ and BMAL1^GTΔC^ protein levels (S3A Fig). Furthermore, no difference in CLOCK level was observed among the different mouse genotypes, consistent with its mRNA patterns (Figs 3B and 4 and S3A Fig).

### Disrupted circadian rhythms at molecular and cellular levels

**Fig 5:**
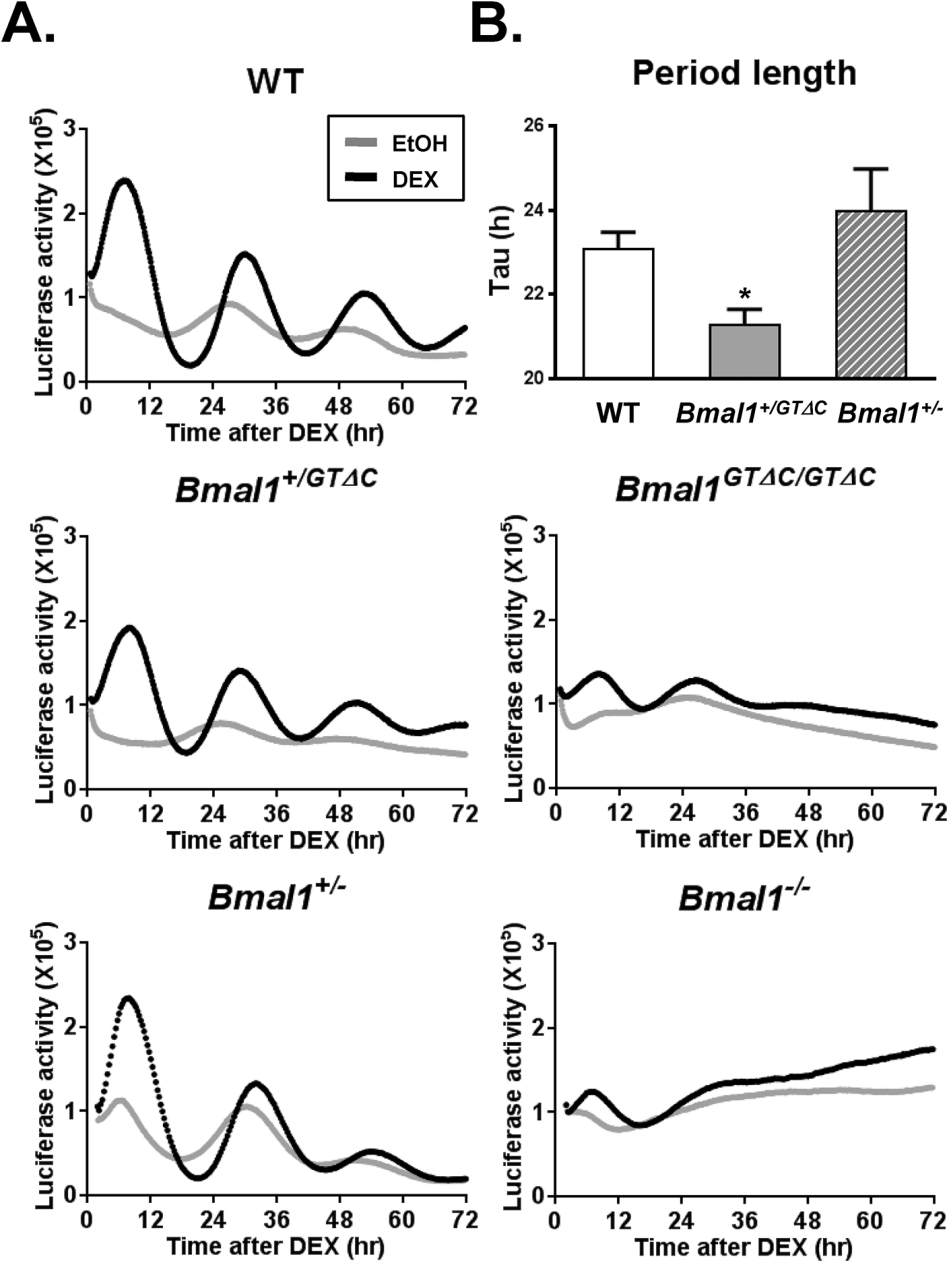
The circadian profiles of MEFs derived from WT, *Bmal1* ^*+/GTΔC*^, *Bmal1* ^*GTΔC/GTΔC*^, *Bmal1* ^*+/-*^ and *Bmal1* ^*-/-*^ mice. **(A)** The representative real-time luminescence profiles of each genotype. Cells were synchronized by 2hr treatment of DEX and monitored by the real-time bioluminescence device. (B) FRP of WT, *Bmal1* ^*+/GTΔC*^ and *Bmal1* ^*+/-*^ cells. Asterisks indicate significant differences (*p<0.05) compared with WT mice. Data are represented as the mean ± S.E.M. (n = 3).

**Fig 6:**
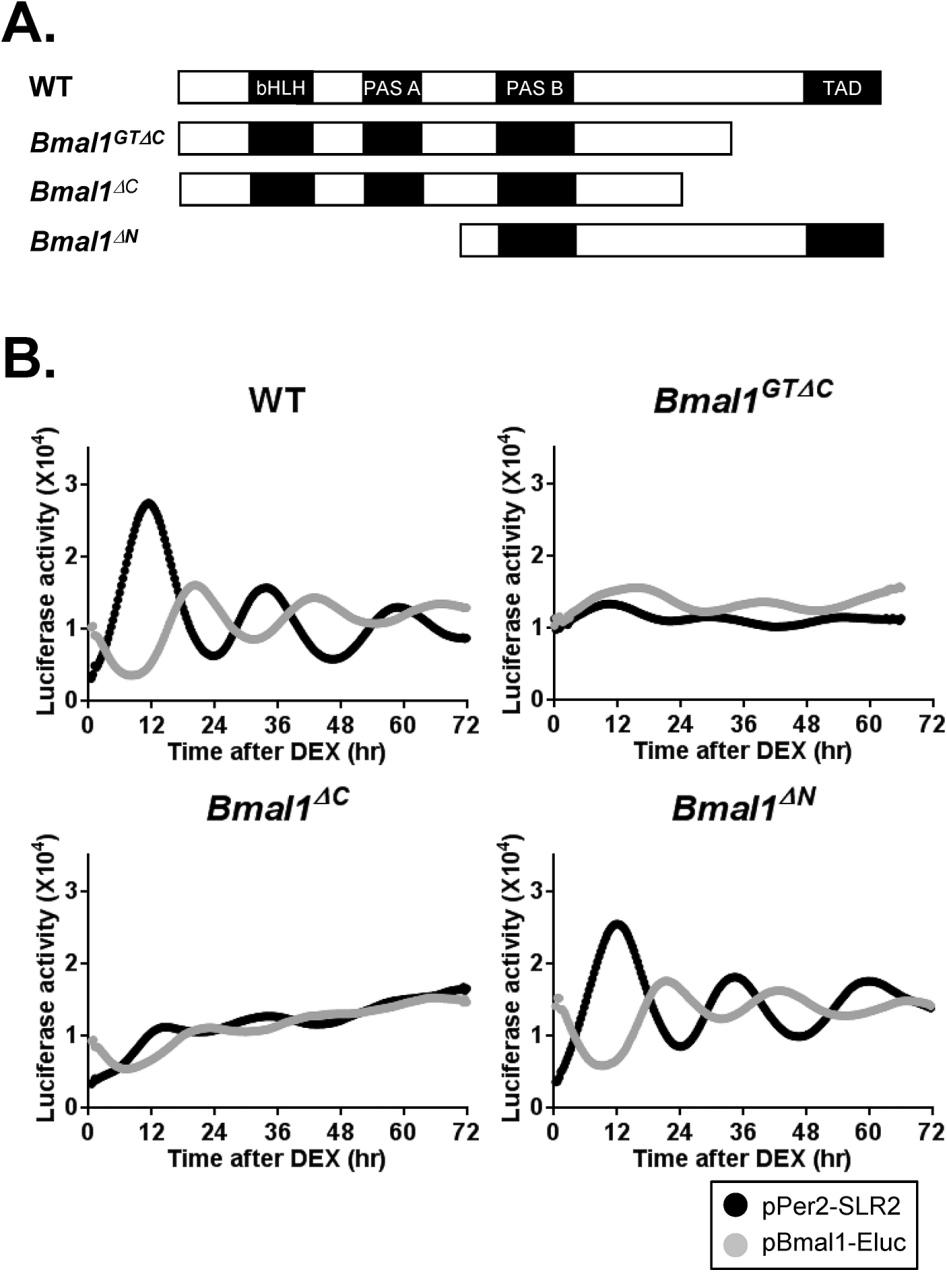
The effects of C-terminal region of *Bmal1* on the molecular circadian rhythm. (A) The structures of BMAL1^wt^ and Bmal1 mutant constructs, Bmal1^GTΔC^, Bmal1^ΔC^ and Bmal1 ^ΔN^. (B) The representative circadian oscillation profiles of the contructs in (A). The constructs were trasfected to WT MEFs. The luciferase activities of *Bmal1* and *Per2* promoters were distinguished by the wavelength separation method (n = 3).

The above data from locomotor activities, mRNA and protein expressions clearly demonstrated circadian arrhythmicity of *Bmal1* ^*GTΔC/GTΔC*^ mice. To determine whether the arrhythmicity resulted from disrupted molecular clock or deficits at *in vivo* systemic level, we generated *Per2*::*luc* knock-in MEFs harboring WT, *Bmal1* ^*+/GTΔC*^, *Bmal1* ^*GTΔC/GTΔC*^, *Bmal1* ^*+/-*^ and *Bmal1* ^*-/-*^ alleles. Despite the fact that *Bmal1* ^*+/GTΔC*^ mice gradually lost the circadian locomotor rhythm, the rhythms of PER2::LUC expression were maintained. The period length of *Bmal1* ^*+/GTΔC*^ mice was significantly decreased, but there was no change in the amplitude, compared with those of WT and *Bmal1* ^*+/-*^ MEFs (Fig 5B and S3B Fig). As expected, the circadian oscillation in *Bmal1* ^*GTΔC/GTΔC*^ MEFs was disrupted to similar degrees as in *Bmal1* ^*-/-*^ MEFs, indicating cellular defects in rhythm generation (Fig 5). To substantiate these results, we introduced a BMAL1^GTΔC^ expressing vector to WT MEFs, and examined circadian oscillation of *Per2* and *Bmal1* promoter activity by using the dual-color luciferase technique [23]. Using this experimental system, we also tested N- and C-terminal truncated Bmal1 constructs (Fig 6A). The overexpression of *Bmal1* ^*GTΔC*^ and *Bmal1* ^*ΔC*^ clearly disrupted circadian rhythms in WT MEFs, whereas no change was observed in WT and *Bmal1* ^*ΔN*^ transfected cells (Fig 6B). These results indicated that the loss of rhythms in *Bmal1* ^*GTΔC/GTΔC*^ mice resulted from the defective molecular clock machinery at the cellular and molecular level.

### Impaired transcriptional activity and degradation of CLOCK: BMAL1^GTΔC^ heterodimer

**Fig 7:**
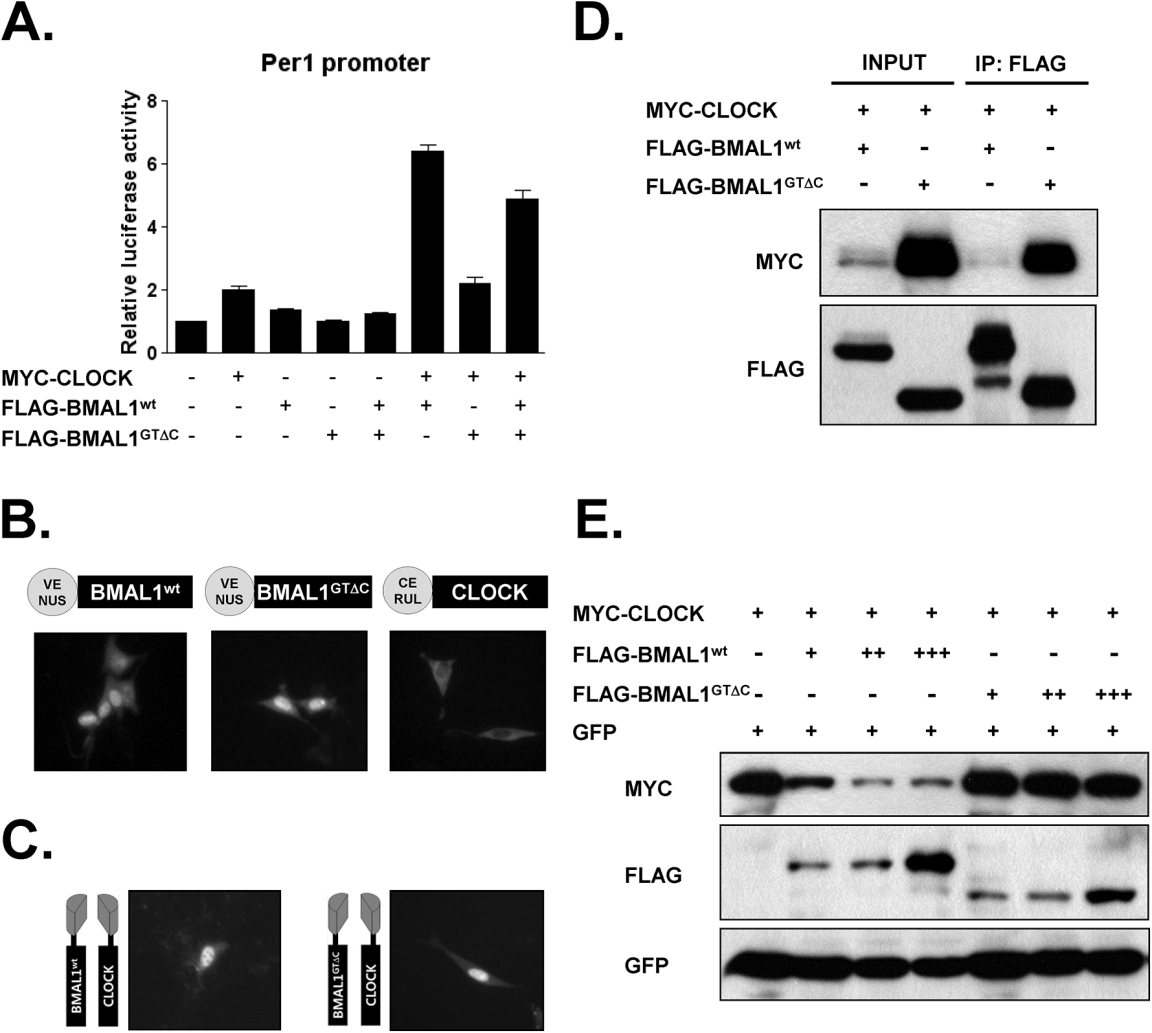
Underlying molecular mechanisms of *Bmal1* ^*GTΔC*^. (A) Effects of *Bmal1* ^*GTΔC*^ on Per1 promoter activity. (B) BMAL1^wt^, BMAL1^GTΔC^ and CLOCK were tagged with fluorescence proteins and the localizations were examined. The heterodimerization and cellular localization of CLOCK:BMAL1^wt^ and CLOCK:BMAL1^GTΔC^ were examined by BiFC assays (C) and IP experiments (D). (E) The dose dependent degradation of CLOCK by BMAL1^wt^ and BMAL1^GTΔC^ (n = 3).

Next, we examined the transcriptional activity of BMAL1^GTΔC^ on *Per1* promoter. Overexpression of BMAL1^GTΔC^ with CLOCK failed to activate *Per1* promoter activity. Interestingly, co-transfection of *Bmal1* ^*wt*^ and *Bmal1* ^*GTΔC*^ partially blocked WT CLOCK:BMAL1 activity, suggesting competition between intact and mutant BMAL1 as reported previously (Fig 7A) [10]. To determine whether the negative role of *Bmal1* ^*GTΔC*^ allele resulted from protein cellular localization, we examined sub-cellular expression of BMAL1^wt^ and BMAL1^GTΔC^ in combination with CLOCK. Either alone or co-expressed with CLOCK, the fluorescent protein tagged BMAL1^GTΔC^ protein showed similar expression with BMAL1^wt^ (Fig 7B). We also did not observe any difference in the dimerization with CLOCK in BiFC assays (Fig 7C). To analyze these results quantitatively, we performed IP experiments. As shown in Fig 7D, CLOCK proteins in BMAL1^GTΔC^-transfected cells showed significantly higher input and IP levels, perhaps as a result of activity-dependent degradation of the CLOCK: BMAL1^GTΔC^ dimer or uneven expression levels. To distinguish these possibilities, we conducted dose-dependency test [25]. The level of CLOCK was rapidly decreased with increased BMAL1^wt^ amount, as reported previously (Fig 7E). However, increased BMAL1^GTΔC^ expression did not attenuate the level of CLOCK. These results are consistent with the compromised transcriptional activation as shown in Fig 7A and the previous report [26].

## Discussion

In the present study, we developed and characterized C-terminal truncated *Bmal1* mutant mice (*Bmal1* ^*GTΔC*^). Molecular and behavioral studies indicated that the circadian rhythms of *Bmal1* ^*GTΔC*^ mice were disrupted in homozygous mutant mice. Interestingly, the heterozygous mutant mice also showed gradual loss of rhythmicity in DD, indicating a semi-dominant negative allele. These results reveal that C-terminal region of BMAL1 plays a key role in generating circadian rhythms *in vivo*.

Circadian mutant animal models have revealed important roles of the circadian clock in physiology, behavior and metabolism [27]. Interestingly, loss of rhythmicity in animal models invariably required homozygous mutations. For example, *Clock* ^*Δ19*^, a classical mammalian circadian mutant allele, led to lengthened and arrhythmic circadian rhythm in homozygous (*Clock* ^*Δ19/Δ19*^), but the heterozygous mutant (*Clock* ^*+/Δ19*^) mice maintain the rhythmicity with lengthened FRP (24.42±0.057, (BALB × B6) F2 progeny) [28]. Here, we report the first circadian “heterozygous” mutant mice showing disrupted rhythmicity in extended DD (Fig 2A).

McDearmon *et al*. (2006) demonstrated that the reduced locomotor activity of *Bmal1* ^*-/-*^ mice was rescued by muscle-specific recovery of *Bmal1*. Furthermore, constitutively expression of its paraolog *Bmal2* in *Bmal1* ^*-/-*^ mice rescued circadian behavior [7]. Interestingly, the total activities of *Bmal1* ^*GTΔC/GTΔC*^ mice were not decreased (Fig 2B). These results suggest that *Bmal1* ^*GTΔC/GTΔC*^ mice retain the partial roles of *Bmal1* on tissue specific functions.

Although CLOCK:BMAL1 is well known to transactivate clock controlled genes, a dual mode of activation and repression has also been suggested in light of the elevated *Cry1* mRNA levels in *Bmal1* ^*-/-*^ mice [29]. In addition to CLOCK:BMAL1, *Cry1* expression is also under regulation of REV-ERBs When REV-ERBs were deleted, the *Cry1* mRNA was reduced [30, 31]. Furthermore, the ChIP-seq experiments revealed that REV-ERBα and β bind to the 5’ upstream region of *Cry1* gene [32]. Consistently, *Bmal1* ^*GTΔC/GTΔC*^ mice showed decreased *Rev-erbα* and increased *Cry1* mRNA expressions, indicating that these mice also employ similar mechanisms of *Bmal1* ^*-/-*^ mice in regulating *Cry1* expression. Surprisingly, we found significantly decreased mRNA levels of *Dbp* and *Rev-erbα* in the heterozygous mutant mice. Considering no significant change in *Per1* and *Per2* mRNA expression between WT and *Bmal1* ^*+/GTΔC*^ mice, these unexpected results indicate that the circadian transcriptional network was disrupted in *Bmal1* ^*+/GTΔC*^ mice (Fig 3B). However, there are still a functional WT allele in *Bmal1* ^*+/GTΔC*^ mice which can sustain rhythms for several days in DD. The abnormal circadian transcription including normal circadian *Per1/2*, decreased *Rev-erbα/Dbp* and increased *Cry1* expression, in *Bmal1* ^*+/GTΔC*^ mice may exacerbate the molecular feedback loop over time and lead to gradual rhythm loss of *Bmal1* ^*+/GTΔC*^ mice under DD. In these contexts, *Bmal1* ^*GTΔC*^ mice represent an excellent model for evaluating the complex interactions of circadian transcriptional network [33].

The roles of the BMAL1 C-terminal region in the circadian clock have been characterized *in vitro*. Collectively, CRY1 and coactivators competitively bind to the transactivation domain (TAD) of BMAL1 encompassing residues 579–626. Deletions, site-directed mutation and replacement of the BMAL1 TAD demonstrated that the TAD is important for both activation and repression of circadian transcriptional activity and plays a crucial role in the generation of circadian rhythms [10, 11, 14]. The previous paper demonstrated that competative binding of CRY1 and CBP (p300) to the TAD generates circadian cycling. As expected, our mutant mice express truncated BMAL1^GTΔC^ without half of the G domain and the entire H domain including the TAD [11]. Furthermore, our mutant mice express BMAL1^GTΔC^ protein which heterodimerize with CLOCK (Fig 7). Compared with the null mutant mice, BMAL1^GTΔC^ also enables investigation of the circadian interaction between the TAD and other circadian transcription factors. Therefore, our *Bmal1* ^*GTΔC*^ mice provide important *in vivo* evidence for the rhythm-generating function of the TAD and an excellent system for further investigations of the circadian control of transcriptional activities.

However, the mechanism underlying the gradual loss of circadian behavior in *Bmal1* ^*+/GTΔC*^ mice remains unknown. As shown in Fig 7E, CLOCK protein levels were not decreased by overexpressed BMAL1^GTΔC^. Previous studies on the degradation dynamics of CLOCK and BMAL1 proteins suggested that CLOCK:BMAL1 activation and proteasomal degradation are tightly linked [25, 26, 34–36]. Stabilized CLOCK: BMAL1^GTΔC^ without transcriptional activty may perturb the balance of BMAL1^wt^ and BMAL1^GTΔC^ expression level in *Bmal1* ^*+/GTΔC*^ mice (Fig 7). However, we did not observe any significant difference in the CLOCK and BMAL1 expression levels among WT, *Bmal1* ^*+/GTΔC*^, *Bmal1* ^*GTΔC/GTΔC*^ and null mice (Fig 4 and S3A Fig). Complex regulatory machanisms may impinge on mRNA and protein levels in the gradual loss of rhythmicity in *Bmal1* ^*+/GTΔC*^ mice, e.g., decreased *Rev-erbα* mRNA, poly(A) tail from the gene trap vector and stabilized BMAL1^GTΔC^ protein. Further investigations into the arrythmic bahevior transition and unusual mRNA expression of *Bmal1* ^*+/GTΔC*^ mice should be conducted.

Although PER and CRY are known as repressors of the CLOCK:BMAL1 complex, recent ChIP-seq data showing distinct spatio-temporal occupancy of CRY1 related to PERs and CRY2 have revised the roles of CRY1 [37]. Furthermore, several recent studies have highlighted potential non-repressive roles of PERs and CRYs on the CLOCK:BMAL1 complexes [38, 39]. As previously reported, the C-terminal region of BMAL1 determines the balance between circadian transcriptional activation and suppression by competitive binding of CRY1 and CBP (p300) [10, 11]. In this context, *Bmal1* ^*GTΔC*^ mice constitute an excellent animal model system for investigating the binding dynamics of PERs and CRYs on CLOCK:BMAL1.

Taken together, we have developed and characterized a novel circadian mutant mice, *Bmal1* ^*GTΔC*^ that exhibited arrhythmicity but otherwise distinct molecular perturbation compared to *Bmal1* ^*-/-*^ mice. Further studies of this mutant will provide crucial insight into molecular mechanisms of the circadian clockwork.

## Supporting Information

S1 Fig The sequences of genomic DNA, mRNA and protein of *Bmal1* ^*GTΔC*^ mice. (A) The structure and insertion site of the gene trap vector on the DNA region. Additional 7bps (underlined) and followed En2 intron (bold) were inserted on the 18^th^ intronic region of Bmal1. (B) The mRNA and protein structures. The truncated *Bmal1* mRNA was linked to *En2* and the following coding sequences. The translated protein was cleaved by the self-cleaving sequence.
(TIFF)

S2 Fig Representative actograms of *Bmal1* ^*+/GTΔC*^ and *Bmal1* ^*-/GTΔC*^ mice. (A) Long term monitoring of wheel running activity in *Bmal1* ^*+/GTΔC*^ mouse that showed circadian rhythms for more than 40 days. (B) Wheel running activity of *Bmal1* ^*-/GTΔC*^ mouse. (C) FFTs of each genotype. The FRPs of WT and *Bmal1* ^*+/-*^ mice were calculated from the data of initial 10 days in DD. Those of *Bmal1* ^*+/GTΔC*^ mice were divided as two different periods. “First” indicates the initial 5 days when the mice were in DD and “Last” indicates the interval of 5 days before losing their rhythms. (D) Effects of genotypes on total activity of wheel running activity in LD. Asterisks indicate significant differences (***p<0.001) compared with WT mice. Data are represented as the mean ± S.E.M. (n = 4~8 per group).
(TIFF)

S3 Fig Comparison of CLOCK and BMAL1 expression levels and the amplitude changes in WT, *Bmal1* ^*+/GTΔC*^ and *Bmal1* ^*+/-*^ MEFs. Representative expression levels of CLOCK and BMAL1. The liver samples of indicated genotypes were collected at two circadian time points and analyzed in one blot. (B) Amplitudes of WT, *Bmal1* ^*+/GTΔC*^ and *Bmal1* ^*+/-*^ cells. Data are represented as the mean ± S.E.M. (n = 3).
(TIFF)
