# Supplementary figures and images for: A Novel Bmal1 Mutant Mouse Reveals Essential Roles of the C-Terminal Domain on Circadian Rhythms
Source: PLoS One. 2015 Sep 22;10(9):e0138661. doi: 10.1371/journal.pone.0138661 (PMC4578957; doi:10.1371/journal.pone.0138661)

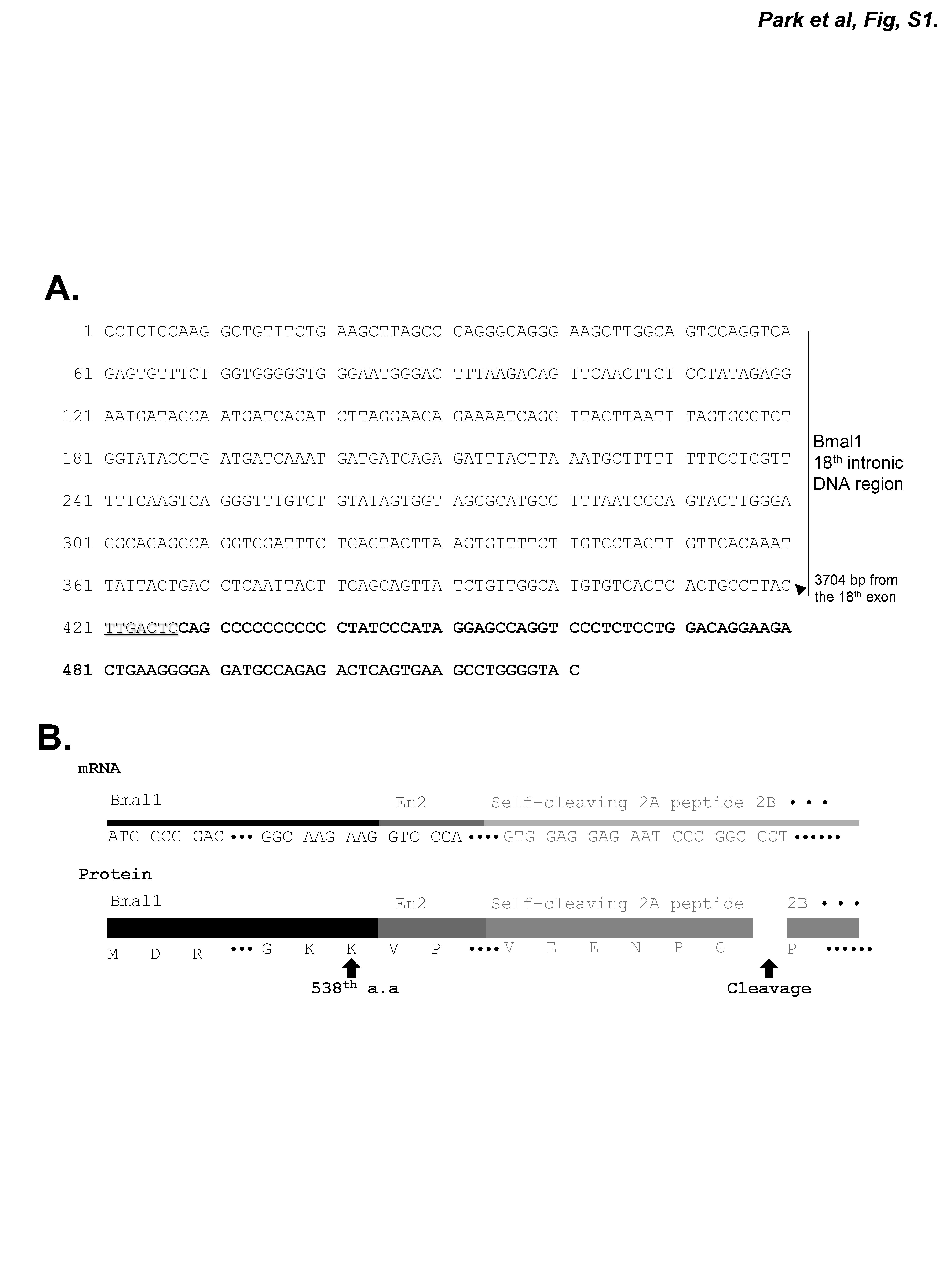

Supplement: S1 Fig — (A) The structure and insertion site of the gene trap vector on the DNA region. Additional 7bps (underlined) and followed En2 intron (bold) were inserted on the 18th intronic region of Bmal1. (B) The mRNA and protein structures. The truncated Bmal1 mRNA was linked to En2 and the following coding sequences. The translated protein was cleaved by the self-cleaving sequence. (TIFF) [file pone.0138661.s001.tiff]

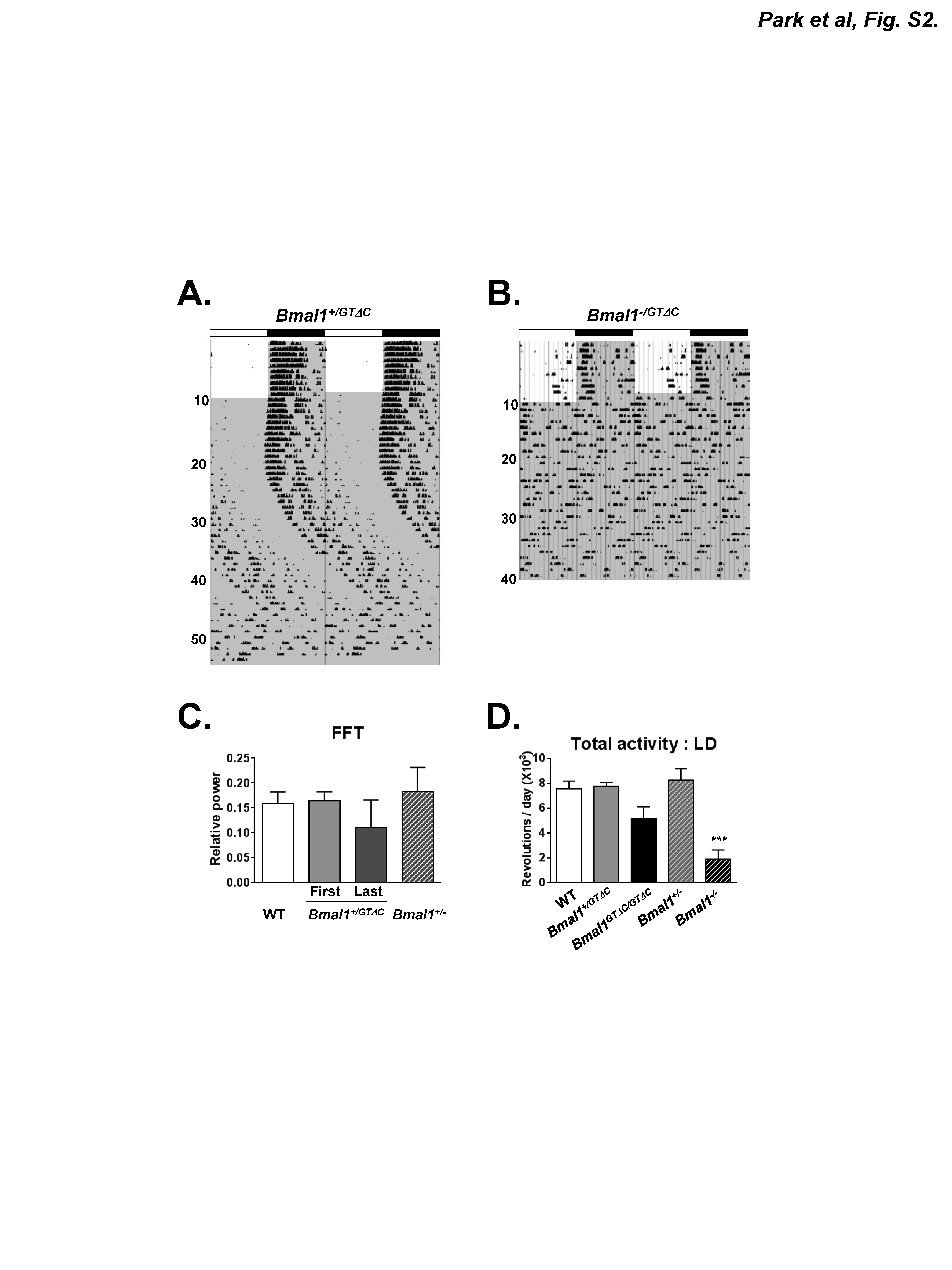

Supplement: S2 Fig — (A) Long term monitoring of wheel running activity in Bmal1 +/GTΔC mouse that showed circadian rhythms for more than 40 days. (B) Wheel running activity of Bmal1 -/GTΔC mouse. (C) FFTs of each genotype. The FRPs of WT and Bmal1 +/- mice were calculated from the data of initial 10 days in DD. Those of Bmal1 +/GTΔC mice were divided as two different periods. “First” indicates the initial 5 days when the mice were in DD and “Last” indicates the interval of 5 days before losing their rhythms. (D) Effects of genotypes on total activity of wheel running activity in LD. Asterisks indicate significant differences (***p<0.001) compared with WT mice. Data are represented as the mean ± S.E.M. (n = 4~8 per group). (TIFF) [file pone.0138661.s002.tiff]

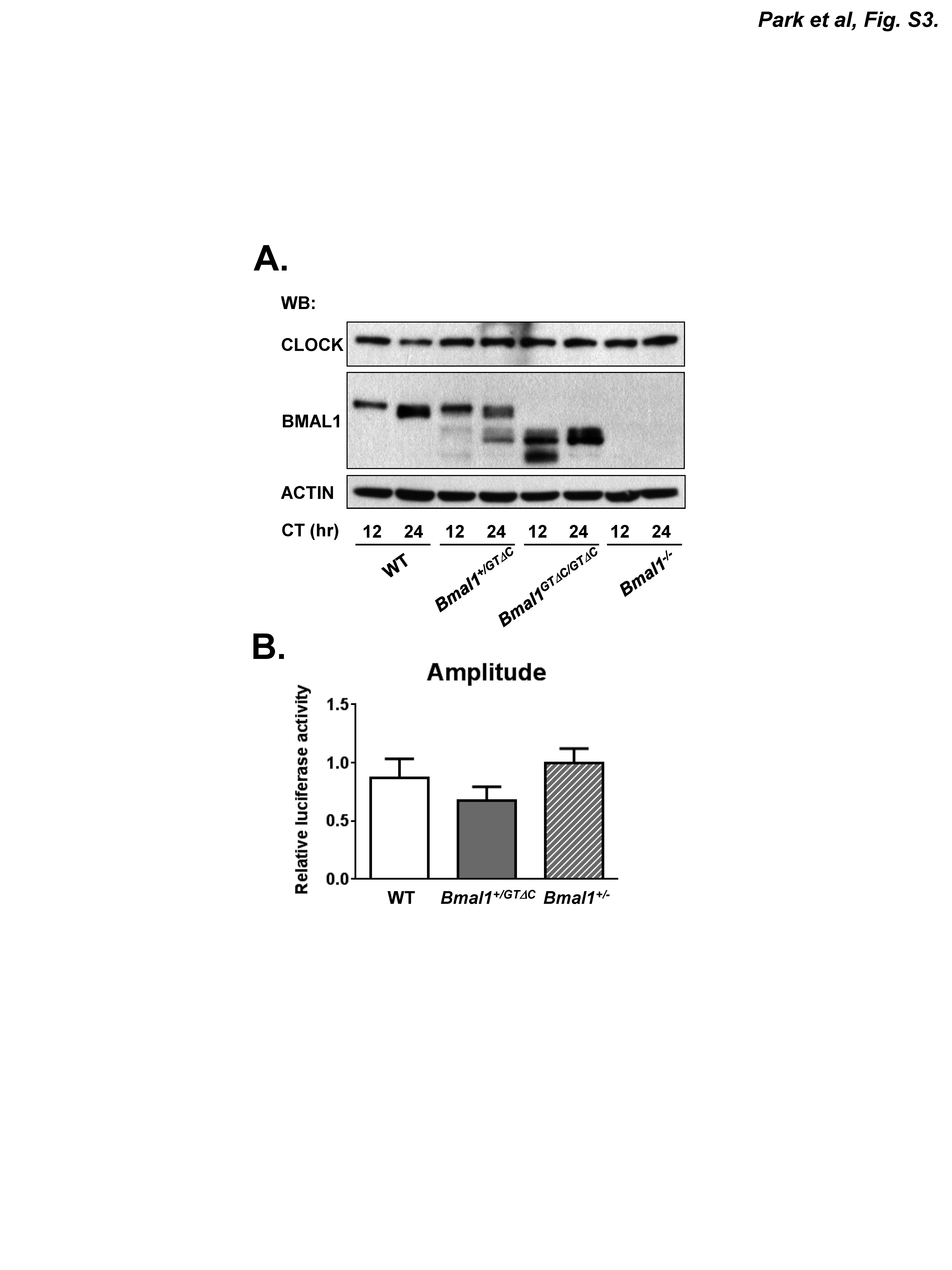

Supplement: S3 Fig — Representative expression levels of CLOCK and BMAL1. The liver samples of indicated genotypes were collected at two circadian time points and analyzed in one blot. (B) Amplitudes of WT, Bmal1 +/GTΔC and Bmal1 +/- cells. Data are represented as the mean ± S.E.M. (n = 3). (TIFF) [file pone.0138661.s003.tiff]
